# Study on safe crossing in the dry season and an anti-floating drainage scheme in the wet season: a case study in Guiyang, Southwest China

**DOI:** 10.1038/s41598-024-52473-x

**Published:** 2024-01-24

**Authors:** Fangzhou Ren, Ning Liu, Cong Zhang

**Affiliations:** College of Civil Engineering, Guizhou University, Guiyang, 550025 China

**Keywords:** Civil engineering, Environmental impact

## Abstract

Karst formations in Southwest China are significantly developed. With the increase in tunnel construction year by year, constructing tunnels in areas of karst geology is inevitable. There are great safety risks associated with exposed karst caves, and the water inside the caves can seriously impact the tunnel structure and its filling materials. Traditional cave disposal technology mainly focuses on safe crossing treatment at the construction stage; however, problems such as backfill collapse and floor floating caused by karst water erosion during the service period are not considered. Therefore, proposals for a new construction scheme to ensure the stability and safety of the tunnel during the service period are urgently needed. Using Huangchongyan Tunnel as an example, we propose a safe crossing scheme of ‘plate–pile–bedrock’ for karst caves, based on a comparison of karst cave treatment schemes at home and abroad. In addition, considering the impact of karst water on the tunnel, we developed a ‘bottom to top’ reverse drainage structure, which solved the problem of floating during tunnel service. In our study, we developed a full life cycle disposal scheme to enable safe passage through tunnels in karst caves, providing a reference for the design and construction of similar projects.

## Introduction

China has the widest distribution of karst landforms in the world, covering one-third of the country’s land area at 344.4 million square kilometers^1^. The Yunnan, Guizhou, Guangxi, and Guangdong provinces in Southwest and Southeast China are the world’s largest karst regions^2,3^. The karst area of Guizhou Province is 110,875 km^2^ and is mainly distributed in the northwest and northeast, accounting for 28.14% of the total area^4–6^. In recent years, China has built a number of high-speed rail tunnels, some of which have encountered huge karst caves^7,8^. The large volume of the cave, the complex nature of the filling material, and the active groundwater affect the safety of the tunnel. Methods for safely passing through karst caves are the main problem in tunnel construction. Scholars have extensively researched how to accurately evaluate the karst caves encountered. Liu and Li et al. used geophysical exploration techniques to analyse tunnels’ geological and hydrological conditions and established a detailed model of karst systems. The detection results are basically consistent with the on-site investigation results^9,10^. Verdet and Jiang et al. established a geological conceptual model describing the origin of karst caves from a geological perspective using three-dimensional geostatistical modelling methods^11–13^. The above research guides disaster prediction and prevention in karst tunnel construction.

When a tunnel inevitably encounters a karst cave, it is necessary to treat the cave accordingly. Li summarised and proposed a new treatment method and construction technology for water gushing from mining pipes in a karst area. This method also has a guiding significance for the karst pipeline’s tunnel construction^14^. Yuan, Liu, et al. introduced detailed construction procedures for tunnel curtain grouting to prevent water- and mud-gushing disasters. They presented optimised grouting parameters, material selection and evaluation^15,16^. Using Wuhan Metro Line 6 as the research object, Wang proposed a series of countermeasures to solve mud gushing and local ground collapse problems in the karst area. Their results showed that the study area successfully avoided water intrusion and controlled surface deformation^17^. Yang proposed a new controllable grouting method and two corresponding grouting materials, addressing the problem that the shield tunnel for Changsha Metro Line 3 must pass through an underwater karst area within the Xiangjiang Water Conservancy Protection Area^18^.

At present, most research focuses on measures for dealing with small- and medium-sized karst caves (i.e., caves that are less than 3 m high), whereas research on tunnelling through larger karst caves is relatively rare. Most of the existing measures for disposing of karst caves focus on safe crossing treatment at the construction stage, but lack any discussion of the stability and safety of tunnels during service. Using Huangchongyan Tunnel as an example, we propose a safe crossing scheme of ‘plate–pile–bedrock’ for karst caves, which compares karst cave treatment schemes at home and abroad. In addition, we developed a ‘bottom to top’ reverse drainage structure considering the impact of karst water on the tunnel. This method solved the problem of floating on the floor during tunnel service. In our study, we developed a full life cycle disposal scheme for the tunnel to safely pass through the karst cave.

## Treatment schemes for karst caves

Considering the particularity of karst caves encountered during tunnel construction and their complex geological conditions, scholars have summarised the characteristics of representative karst tunnels and proposed specific disposal measures based on engineering and construction experience at home and abroad.

To safely cross the karst caves encountered in tunnel construction, scholars at home and abroad have conducted extensive research. Using the Yiwan Railway as an example, Fan proposed a pile foundation, namely, a cap scheme, steel pipe pile reinforcement, and other methods, for karst caves with different filling conditions. This treatment method provides a reference for tunnels to safely pass through karst caves in the future^19^. In order to demonstrate the rationality of the backfill parameters and the effectiveness of the supports when a tunnel passes through a large karst cave, Wang et al. used the finite element software FLAC3D for numerical analysis. The field monitoring results showed that the optimal backfill parameters can effectively reduce the displacement around the tunnel. Their study provides a reference for the design and construction of other projects in the future^20^. Using the largest karst cave in the history of China’s railway construction as an example, Zheng introduced comprehensive treatment methods for a giant karst cave, including backfilling of the cave, reinforcement of the cave roof, reinforcement of both sides and the bottom of the tunnel, backflow interception of the underground river, and bridge construction in the tunnel^21^. Using the Laobishan Tunnel of the Chengdu‒Kunming Railway as an example, Miao et al. proposed a reasonable crossing scheme given the problems encountered during tunnel construction. According to the investigation results, they decided to adopt an arch bridge with a protective structure to cross the karst cave. Their proposed solutions and recommendations can guide similar projects^22^. Based on the treatment and construction process of Naqiu Tunnel’s corridor cave, Chen introduced a disposal method for pile caps and retaining walls located in the steep slope section that effectively suppressed the influence of surrounding rock bias^23^. Using the Vrata tunnel on the Zagreb–Rijeka highway as an example, Garašić et al. proposed a scheme of a bridge crossing the karst cave according to the cavern’s size, shape, position and hydrogeological parameters within the karst system. In addition, the cavern’s vault had to be reinforced and stabilised, as the overburden was very thin. The cavern’s rehabilitation in the Vrata tunnel was a unique undertaking, and the bridge (without piers) is the longest cavern bridge in the world^24^. Using the Gavarez Tunnel in Spain as an example, Alija et al. proposed ground improvement techniques for pre-support and excavation edging, given the problems encountered during tunnel construction. By strengthening the backfill, the tunnel can pass through the cave safely. Their proposed solutions and recommendations can guide similar projects^25^. Based on the treatment and construction process of Yujingshan tunnel, Xie et al. present the unique structures of a bridge supporting railway tracks wrapped by tunnel lining and the settlement control of the tunnel crossing a massive rockfill in the giant cave. Their proposed solutions and recommendations can guide similar projects^26^. Using Shaoxing Metro Line 1 as an example, Huang et al. implemented targeted reinforcement measures at different risk sections based on previous geological exploration results. Their proposed tunnel-crossing scheme ensures safety during construction and operation^27^. Based on the Swabian Jura high-speed railway, Kielbassa introduced karst area survey results for selected parts of the new line from Stuttgart to Ulm and proposed different treatment measures^28^. Using the Cheng-gui Railway as an example, Chen et al. established a numerical simulation model for the backfilling of a large karst cave through a large cross-section tunnel. They presented optimised backfilling parameters, material selection, and evaluation^29^. Wentao and Xu et al. used the geological conditions of karst areas in Wuhan and the design of some subway tunnels as examples, analysed potential problems in karst areas, and discussed disposal plans for subway tunnels passing through karst caves in this area^30,31^. Based on the spatial relationship between tunnels and caves, Shi et al. summarised relevant treatment techniques and proposed a spatial decomposition method for large caves. This method can provide a reference for selecting the treatment technology^32^. Wang used the giant karst cave of Qianchang Railway’s high mountain tunnel as a research object. Considering construction, operation safety, the economy, and other factors, he determined the ‘backfill cave + upper grouting reinforcement’ karst cave treatment scheme. His final results met the design requirements and showed that the backfilling treatment of the cave was stable, and the settlement after construction was controllable^33^. Table 1 summarises the general disposal methods for tunnels crossing karst caves.

**Table 1:** Crossing scheme of karst tunnel.

Scheme	Disposal method	Advantages	Disadvantages	Sketch
Bridge crossing karst cave	Reduce the treatment measures for the tunnel bottom, and support the bridge pile foundation with the bottom bedrock after passing through the accumulation	Less treatment measures for tunnel bottom, with stronger applicability	The process is complex, and the construction is difficult; the bearing capacity of the bottom foundation must be high; the pier’s ability to resist rockfall impact is weak	
Pile caps across the karst cave	Instead of the secondary lined invert, the tunnel adopts a concrete cap. The upper part of the pile foundation is connected to the cap to establish the joint crossing of the cave	Quickly resume tunnel construction with mature construction technology	In the rainy season, the groundwater in the karst cave flows freely, causing leakage and safety hazards to the tunnel	
Backfill treatment	Use tunnel excavation material and rubble concrete to backfill the exposed karst cave in layers	Less pollution to the environment, rapid recovery of tunnel construction, and low-cost and mature construction technology	Caulk treatment in large karst caves has higher requirements and poor applicability	
Composite foundation scheme	Reinforce, replace, and set reinforcement materials for part of the soil in the original foundation	The treated foundation has high strength and good structural stability	Controlling the construction quality is difficult, and the cost is high	

To reduce the adverse effects of water on the tunnel structure, extensive research has been conducted on waterproofing tunnels and drainage structures. Considering the shortage of waterproof boards in traditional tunnel drainage systems, Zhang proposed a composite waterproof board to add to the drainage plate between the tunnel waterproof board and the secondary lining. A composite waterproof board comprises capillary drainage plate geotextiles or geotextiles that have good adjustable ability, strong blockage prevention ability, and convenient maintenance^34^. To solve the problem where conventional drainage prevention and drainage schemes cannot meet the requirements of water-rich karst tunnels, Zhao proposed three optimisation schemes for drainage and prevention by combining numerical simulation and model tests. The results show that the central drainage ditch at the bottom of the invert significantly affects water pressure reduction^35^. By analysing several historical cases, Luciani et al. highlighted the importance of water preservation and the potential danger of underground excavation for the environment if there are insufficient controls of underground water. They presented a global overview of the techniques used and proposed a risk analysis procedure^36^. Using Guiyang Rail Transit Line 1 as an example, Liu et al. systematically analysed various causes of water and mud penetration and proposed an innovative scheme for tunnel treatment in karst areas involving drainage wells and connecting channels. Consequently, the confined karst water can flow to the surface by itself^37^. Zhou established a two-field fluid‒structure coupling calculation model based on the field monitoring results of the Taidacun tunnel on the China–Laos Railway to optimise the waterproofing and drainage design of tunnels with weak water-cut formations. Zhou also demonstrated the influence of the water level and annular blind tube spacing on the lining water pressure and determined the optimal distance of annular blind tubes in water-rich zones of the tunnel^38^. Using Norwegian rail and road tunnels as examples, Holter introduced a composite tunnel lining system based on a sprayed waterproofing membrane combined with sprayed concrete. This tunnel lining system consists of a waterproof membrane which, during application on the sprayed concrete lining, bonds mechanically to the sprayed concrete on either side. Hence, a continuous, sealing, and non-draining structure from the rock mass to the interior tunnel surface is formed in the walls and crown^39^. Zhang proposed a new drainage structure through numerical simulation and laboratory experiments. In this structure, the circular drainage tube is removed, and a convex shell drainage plate is added between the waterproof plate and the secondary lining. Their results showed that the new drainage system greatly reduces the water pressure in areas prone to blocked drainage structures^40^. Wang researched Wuhan Metro Line 6 and proposed a series of countermeasures for hazard and risk mitigation. The study section successfully avoided water seepage and controlled ground deformation during the whole construction phase^41^. Using the Gongbei tunnel project of the Hong Kong–Zhuhai–Macao Bridge as an example, Zhao studied the design of the waterproof drainage system and the Gongbei tunnel’s detailed structure using indoor and field tests. Zhao’s results showed that an integral waterproofing system combining a grouting ring, pipe top freezing ring, initial lining, waterproof plate, and three-stage lining should be adopted for the Gongbei tunnel^42^. Dammyr proposed a waterproof concept for the future of Norwegian railway tunnels by collecting and analysing the operation experience of tunnels with different waterproof concepts. Their proposed solutions and recommendations can guide similar projects^43^. Using the Longtan Tunnel and Baiyun Tunnel as examples, Ding proposed a composite waterproof drainage system for controlling drainage, including a water-blocking grouting ball, main support, waterproof drainage grid system, and anti-hydraulic lining^44^. Yuan summarised the waterproof requirements and measurements of different tunnels in China as well as the limitations of common waterproofing and drainage methods, such as waterproof lining, drainage systems, and grouting. Yuan also proposed some feasible measures for concrete lining and drainage facilities^45^. Table 2 summarises the general methods of tunnel waterproofing and drainage.

**Table 2:** Treatment scheme for karst tunnel drainage.

Scheme	Applicable conditions	Disposal method	Advantages	Disadvantages
Composite lining	Dry or small water tunnel	Through the waterproof performance of the lining itself to realize the drainage, filling waterproof material between the lining can achieve better drainage effect	Low cost, strong applicability, variety of waterproof materials	The requirements for construction technology are high and generally used together with other schemes
Drainage Waterproofing	Tunnels with large flow or environmental requirements	Laying drainage ditches and blind pipes in advance can prevent tunnel drainage	Strong applicability and significant drainage effect	The construction quality requirements are high, which are easy to block
Grouting Waterproofing	Tunnels with shields or poor surrounding rock properties	Chemical grouting and granular cement grouting can improve the integrity surrounding rock in tunnels and reduce their hydraulic pressure	High safety allows for self-waterproofing of the grouting layer	High cost, difficult-to-control construction quality, poor waterproof effect

Based on the domestic and international excavation and groundwater treatment methods, we have proposed a comprehensive life cycle tunnel treatment method known as “crossing in the dry season and an anti-floating drainage scheme in the wet season”. During the construction phase, we have implemented a top heading support scheme and adopted the filling layer reinforcement method. For flood discharge, we have designed a reverse drainage structure that operates from the bottom to the top. The proposed method aims to address both the challenges posed by groundwater during tunnel construction and the potential risks associated with floating caused by heavy rainfall. By excavating the tunnel in the dry season, we can minimise the impact and facilitate the construction process. In the wet season, when the risk of floating increases, we implement an anti-floating drainage scheme to effectively drain excess water and maintain stability. By integrating these different methods into a comprehensive approach, the full life cycle tunnel treatment method offers a systematic and effective solution for addressing the challenges related to both groundwater and flood risks. By carefully considering the specific conditions and requirements of each phase, we can ensure the successful construction and long-term stability of the tunnel.

## Project overview

### Project site

**Figure 1:**
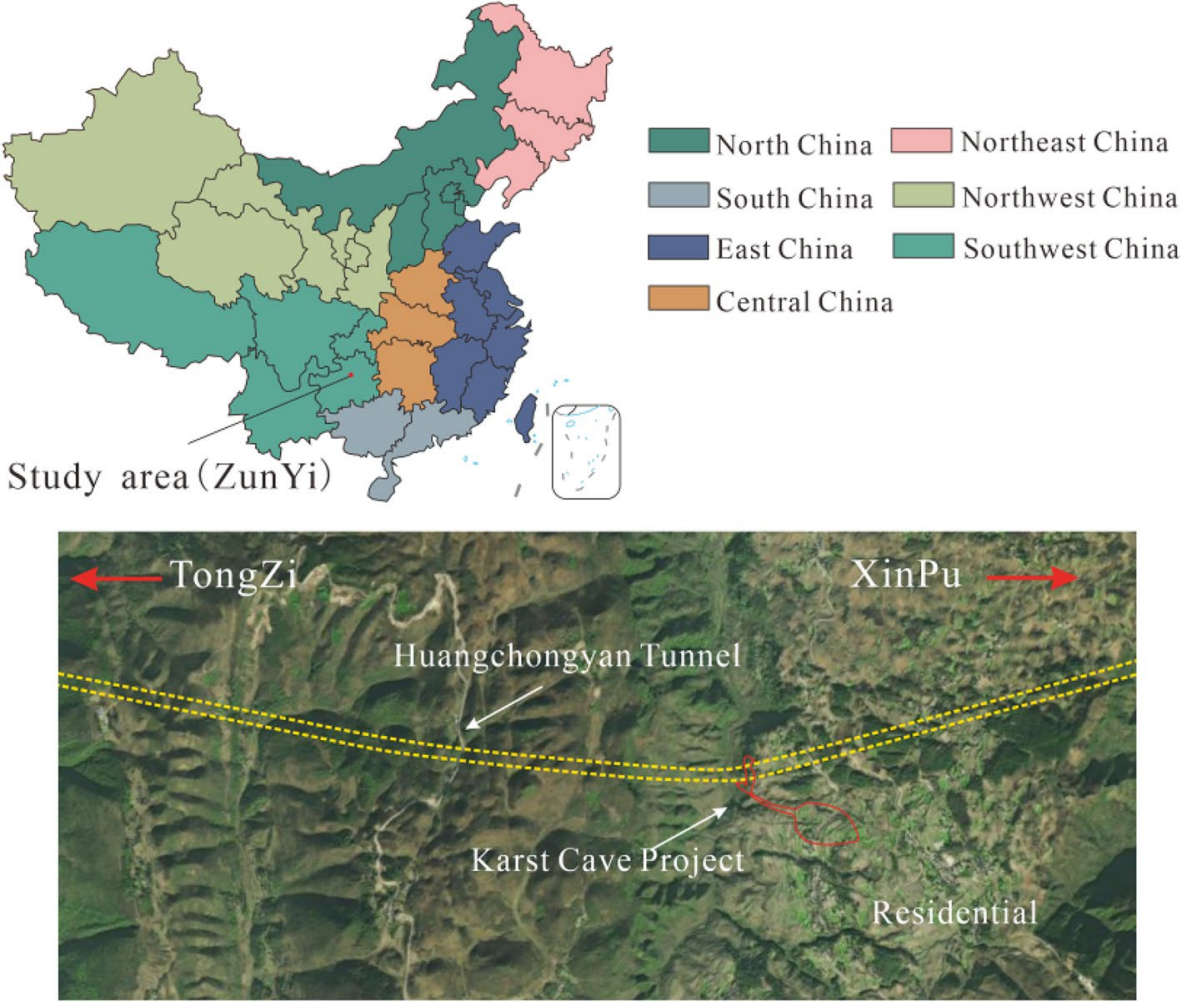
Topographic plan of the proposed engineering.

**Figure 2:**
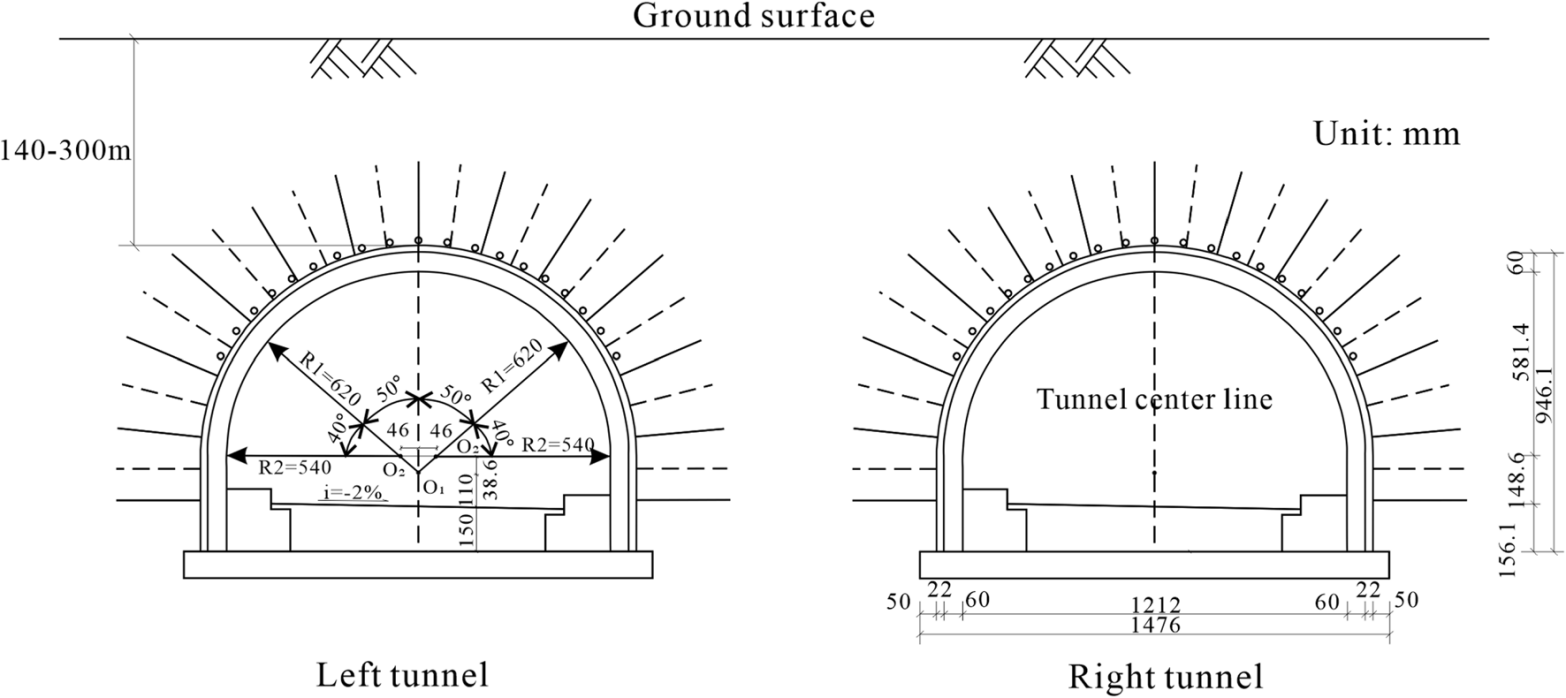
Schematic diagram of cross-section of the tunnel.

The project area is located in Fengba Town, Suiyang County, Guizhou Province. It is part of the dual-line tunnel from Tongzi station to Xinpu station, on the Tongxin Expressway in Guizhou Province. Figure 1 shows the relationship between the tunnel and the surrounding environment. The buried depth ranges between 140 and 300 m. The two tubes are arranged in an east–west configuration. A horseshoe-shaped double-shell lining is used as a permanent tunnel support. The excavation area of a single tunnel is about 114.6 m^2^ (width × height: 12.12 × 9.46 m), as shown in Fig. 2. The total length of the tunnel is about 2210 m. According to the qualitative characteristics of the rock mass and the basic quality index (BQ) of the rock mass, the grade of the surrounding rock of the running tunnel is III and IV^46^. The development of joint fissures is attributed to the influence of the geological structure. The density of the surrounding rock is 1.75–2.67 g/cm^3^. The uniaxial compressive strength, the tensile strength and the elastic modulus of the surrounding rock is 1.45‒40.9 MPa, 0.11‒4.8 MPa, 198, and 0.42‒15.02 GPa, respectively.

### Geological and hydrogeological conditions

#### Topography

The project area is located on the Northern Guizhou plateau, which is part of the Dalou Mountains. The area belongs to the discontinuous erosion geomorphic unit, with undulating mountains and vertical and horizontal valleys. The entrance has a sloping terrain covered by clay layers on the surface and well-developed vegetation. The terrain at the exit end is gentle, and the cave body passes through the ridge. The altitude of the site is between 980.7 and 1446.8 m, with a relative elevation difference of 200–400 m. The terrain slope is generally 20–40, the middle of the terrain is higher, and the east and west are lower. The terrain is shown in Fig. 1.

#### Geological structure

The project area is located in a structurally deformed area. The fractures and folds in the area are mainly north–south. There is a normal fault in the field area, intersecting with the line at YK22 + 065, which significantly influences the tunnel structure. There is no obvious weak interlayer or muddy fill between the structural planes, which are hard structural planes. After multiple geological and tectonic movements, secondary structures have developed due to the influence of regional structures and normal faults. There are many local folds, tectonic tensile joints, and cracks. The rock mass is relatively broken, creating favourable conditions for karst development.

#### Lithology

**Figure 3:**
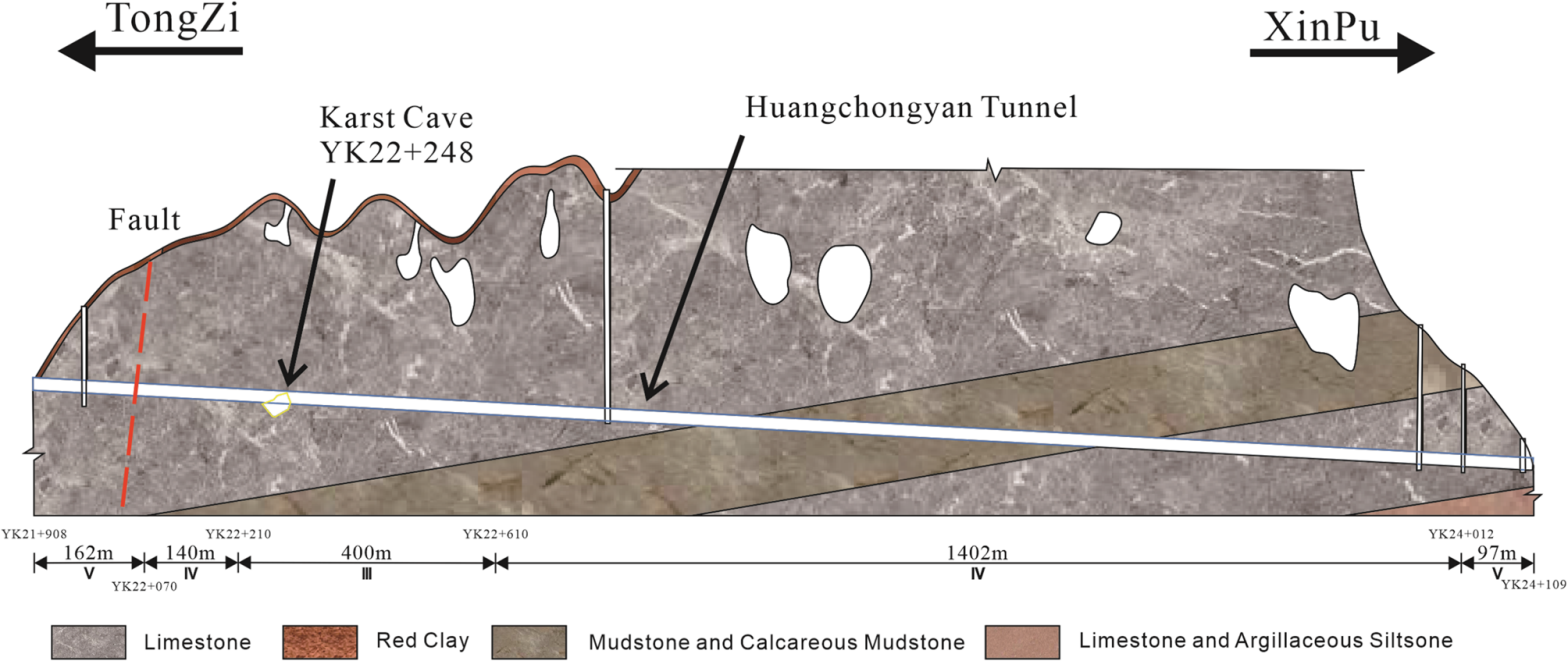
Geological profile of interval tunnel.

Soil conditions at the site were investigated through on-site drilling before construction. Our results showed that the geology at the construction site had mainly Quaternary soil layers and Permian strata, including soft soil, red clay, limestone, carbonaceous shale, mudstone, calcareous mudstone, and silty mudstone. The specific soil descriptions are as follows: (1) Soft soil: brown, soft plastic, relatively pure, and sensitive soil, distributed at the front of the hill flat and mountain grooves, with a filling thickness of about 0–1 m. (2) Red clay: brown-yellow, yellow, and plastic mixed with 5% gravel and homogeneous soil containing a small amount of gravel and breccia, thin in most areas at 0–3 m thick, thick in some areas at 5–8 m thick, mainly distributed on the slope. (3) Limestone: dark grey, strongly weathered, thin to medium-thick layers, aphanitic structure, 0–2 m thick, soft rock, with relatively developed joint fissures and broken rock masses. The quality of geotechnical construction was grade IV soft rock. (4) Carbonaceous shale: brown, dark brown, strongly weathered, thin layer, carbonaceous structure, about 0.1–0.3 m thick, matte, easy to soften with water, with occasionally poor coal quality in the coal line. The quality grade of geotechnical construction was grade IV soft rock. (5) Calcareous mudstone: purplish red, grey-green, strongly weathered, 3–8 m thick, thin to medium-thick layers, argillaceous structure, rock fragmentation, bedding, easy to soften and peel when exposed to water, with the ability to split into sheets along the bedding plane. The quality grade of geotechnical construction was IV soft stone. (6) Silty mudstone: greyish yellow, strongly weathered, argillaceous structure, thin to medium-thick layered structure, and rock fragmentation, with well-developed joints and fissures. The quality grade of geotechnical construction was IV soft stone. Figure 3 shows the geological profile of the interval tunnel. Table 3 shows the rocks’ physical and mechanical parameters in the interval.

**Table 3:** Rock physical and mechanical parameters.

Rock	Density (g/cm^3^)	Uniaxial compressive strength (MPa)	Tensile strength (MPa)	Elastic modulus (GPa)	Poisson ratio u	Angle of friction (°)	Cohesive strength (MPa)
Red clay	1.75	–	–	–	–	8	0.015
Limestone	2.6	40.9	4.8	4.1	0.23	45.0	0.3
Carbonaceous shale	2.67	32.9	4.4	15.02	0.25	32.2	1.36
Calcareous mudstone	2.58	1.53	0.13	0.43	0.23	26.3	0.13
Silty mudstone	2.53	1.45	0.11	0.42	0.23	25.7	0.11

#### Hydrogeological conditions

The area belongs to a subtropical plateau monsoon humid climate zone characterised by abundant rainfall, dry and wet seasons, and a long frost-free period. The precipitation is the most in summer and the least in winter, but it is dry in winter and wet in summer. The annual average number of cloudy days is 245, and the annual average sunshine is 1091.6 h. Due to the large altitude difference, variation of temperature with height is significant.

**Figure 4:**
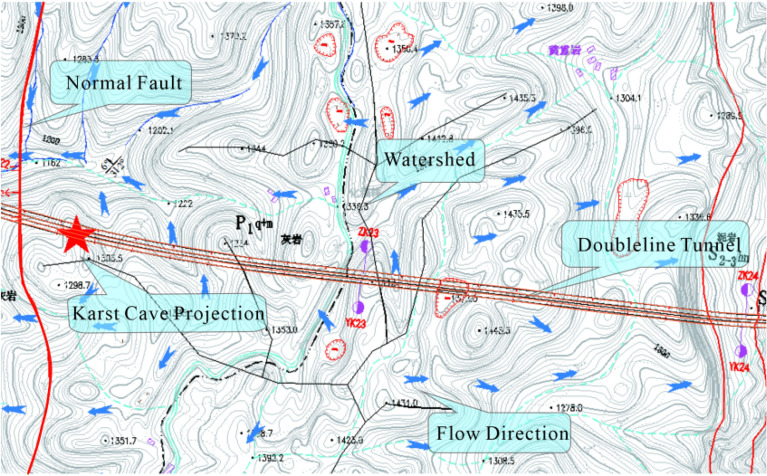
Interval surface hydrogeological map.

There is a minor occurrence of surface runoff within the tunnel region. There is a perennial stream outside the tunnel entrance, with a flow rate of 10 L/s during the inspection in August 2021, which is greatly affected by the rainy season. Figure 4 shows the hydrogeological map of the surface.

Karst water appears 40 m to the right of YK22 + 260 in the karst cave. It flows out of the karst fissure in the form of cascade. The water flows in a small mileage direction with clear water quality. During the survey, the water flow was about 0.5–1 L/S. Another karst water source appears 72 m to the right of YK22 + 300. It flows out of the karst pipeline, with a visibility of about 5 m. The water flows in a small mileage direction with clear water quality. During the survey, the water flow was about 1–2 L/S.

## Crossing the cave

### Description of karst cave encountered

**Figure 5:**
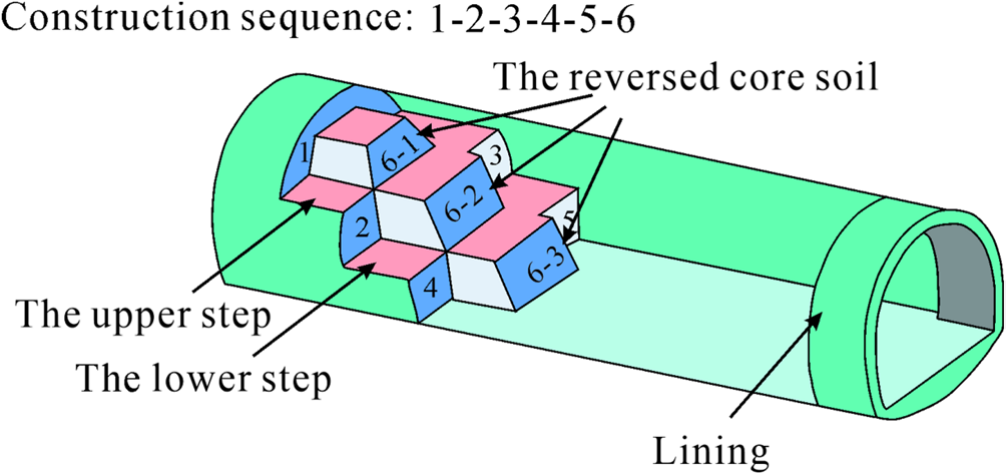
Schematic diagram of bench method.

**Figure 6:**
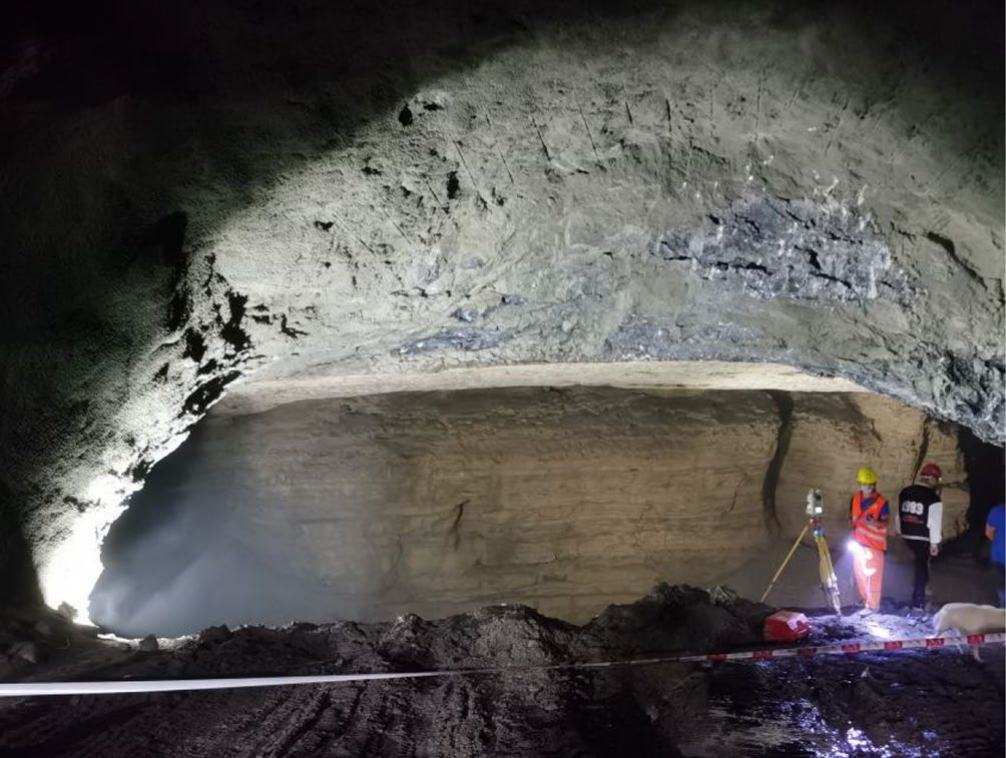
Tunnel face encounters karst cave.

The tunnel was excavated from Tongzi to Xinpu using the bench method. The construction sequence of the bench method is illustrated in Fig. 5, and the excavation is carried out in the order from 1 to 6. Before the karst cave was revealed, no other groundwater was exposed on the nearby surface. The surrounding rock is relatively competent, without leakage or water inflow. On 17 July 2021, the tunnel face of the Tongzi end of the left section was excavated to ZK22 + 228, exposing a large semi-filled karst cave. The sudden encounter with the karst cave led to the collapse of the reserved core soil on the tunnel face, as shown in Fig. 6.

**Figure 7:**
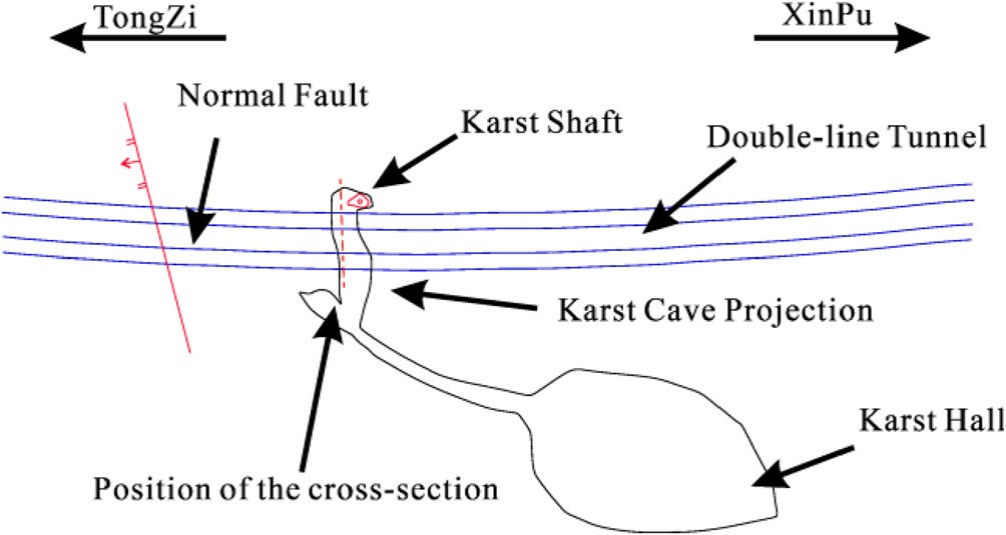
Location of karst cave.

**Figure 8:**
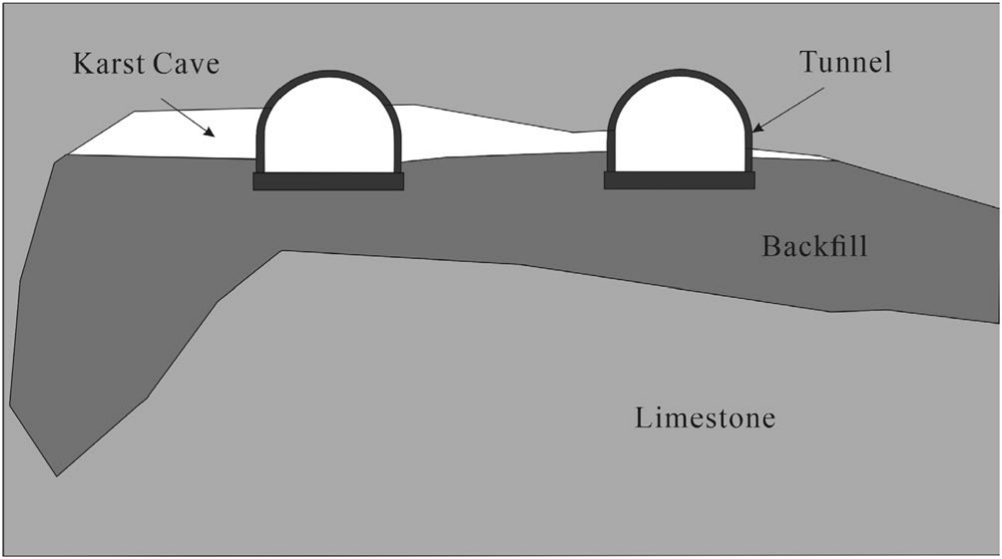
Cross-section of karst cave.

After the large semi-filled cave on the left side of the tunnel was exposed, the construction participants immediately organised an on-site geological survey. The investigation revealed that the cave developed along the rock layer from left to right. The caves intersect almost vertically at ZK22 + 228 ~ ZK22 + 248 (YK22 + 238 ~ YK22 + 2K264), with a lateral length of about 115 m and a height difference of 8–15 m. The extent of the tunnel through the cave is the highest point at the bottom of the cave, developing from left to right downwards. This is shown in Figs. 7 and 8.

**Figure 9:**
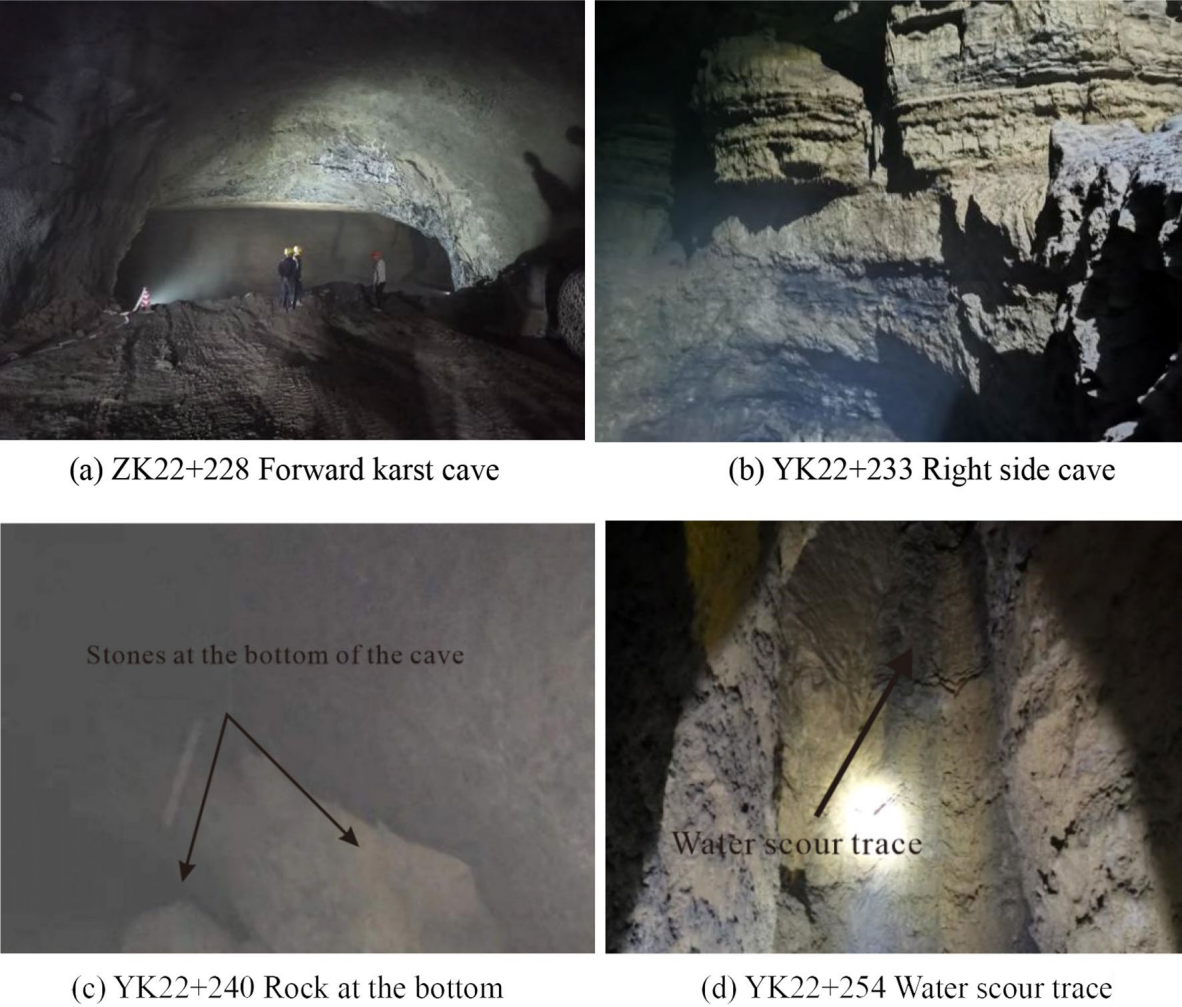
Karst cave in tunnel.

The lithology at K22 + 228 is mainly limestone, with relatively developed joint fissures and broken rock masses. It is a rigid structural plane of general combination, with mud filling between the structural planes. The cave wall is complete, with a smooth surface and obvious traces of water erosion. The karst cave develops downwards at 40 m on the right side of tunnel YK22 + 240, with the deepest point about 10 m from the tunnel floor. At the bottom of the cave there are accumulations of stones. Figure 9 shows the situation of the cave in the tunnel.

**Figure 10:**
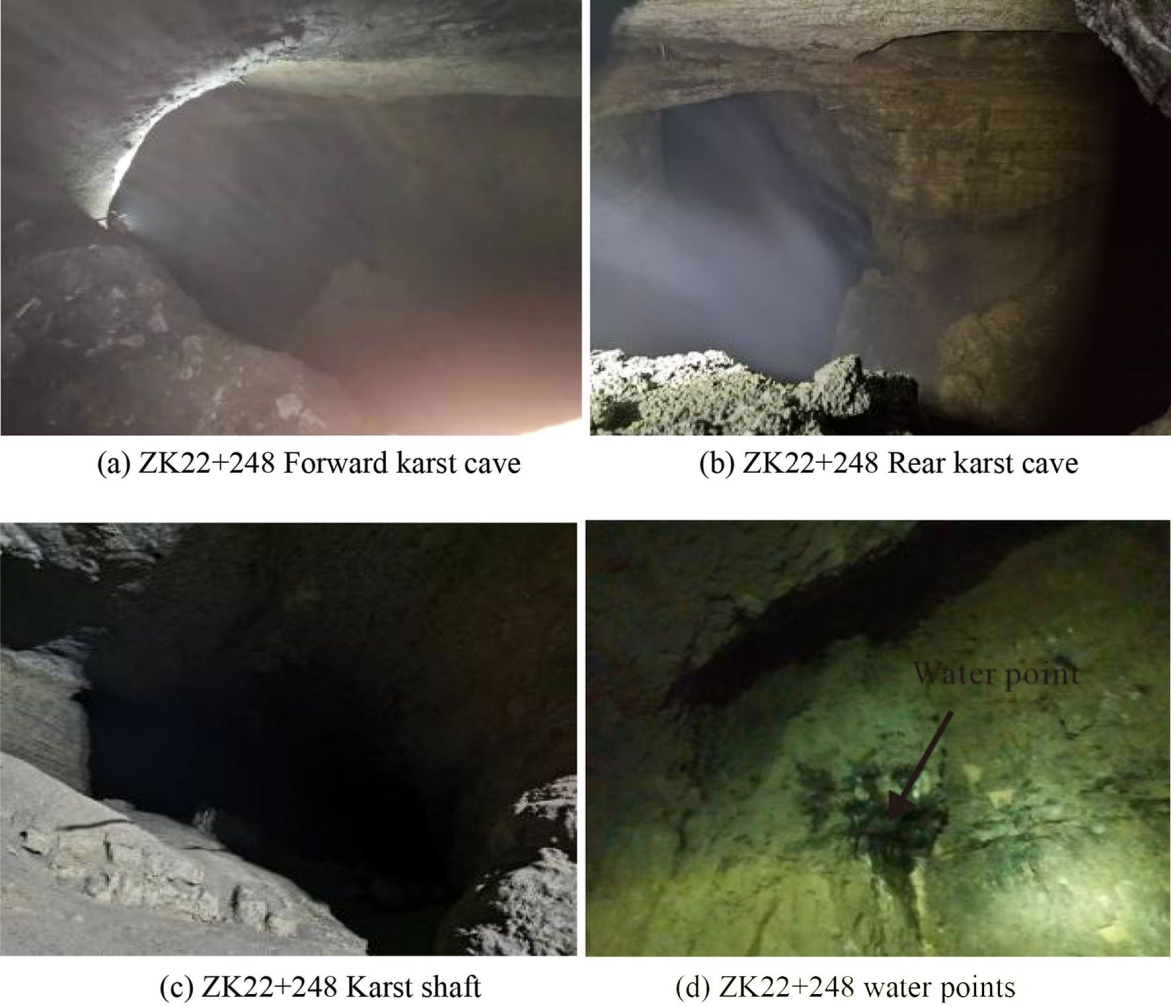
Karst cave in left tunnel.

**Figure 11:**
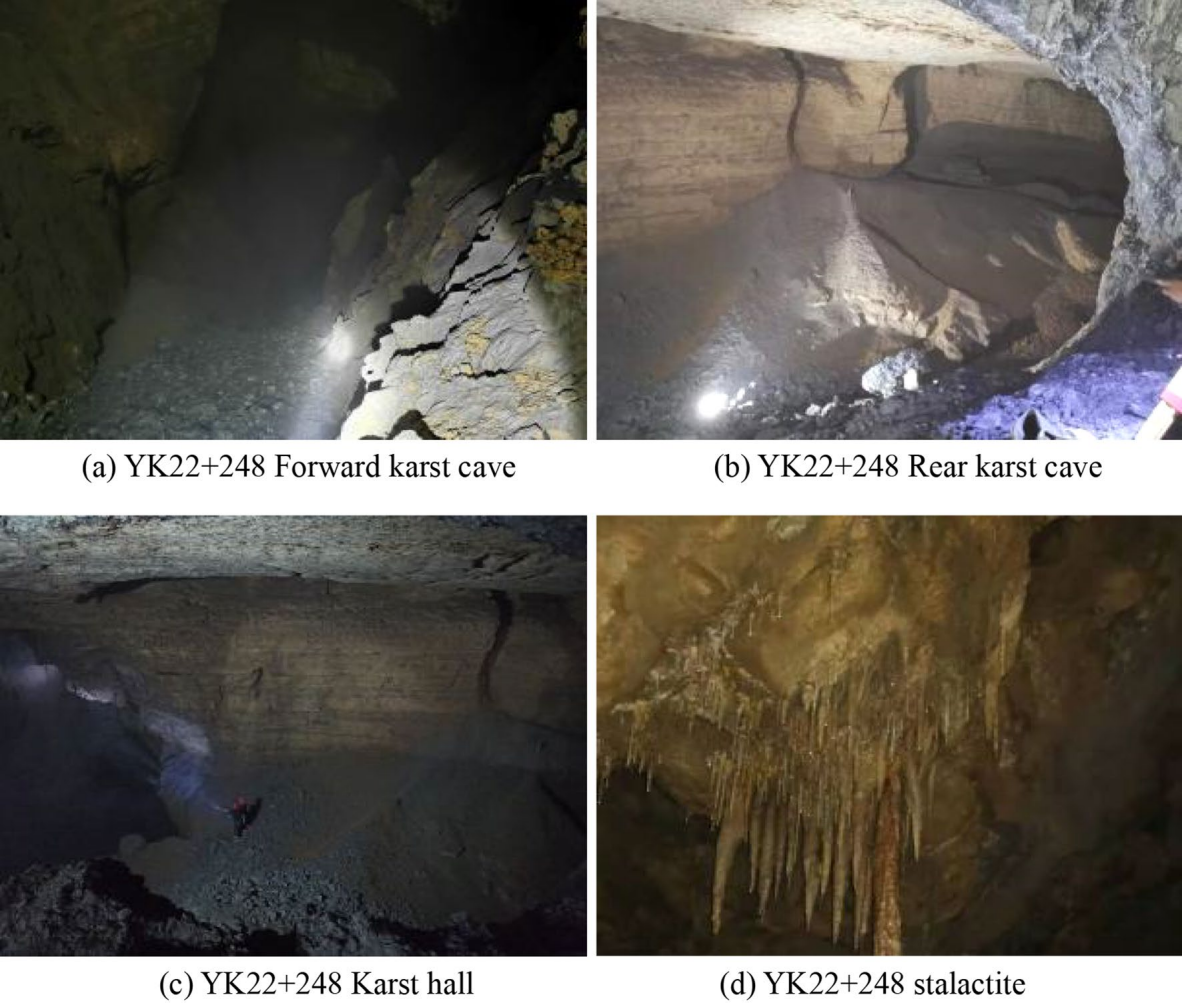
Karst cave in right tunnel.

The cave extends vertically in a funnel shape about 20 m to the left of the left tunnel, with a visible depth of about 45 m, and then continues to develop downward in the form of a groove. Mist emerges from the bottom of the cave. The cave wall is intact, and the surface is rough. The upper part of the wall is dry, whereas the lower part is wet and accompanied by water droplets, as shown in Fig. 10. The angle between the karst cave and the right tunnel is 25°. After 192 m long development, the karst cave gradually turns into a hall. The hall is oval, about 205 m long and 130 m wide, with a height of 20–40 m. The hall has developed under a steep slope, with a deep pool at the lowest point, which had flowing water and was presumed to be a karst river, as shown in Fig. 11.

### Potential causes of complex karst cave

Through our investigation and analysis of the geological conditions in the karst caves, the potential causes of the complex karst cave are as follows:

#### Complex regional structures

As previously mentioned, the engineering area is located within a tectonic deformation zone. Due to the influence of regional structures and faults, the rock mass is relatively broken, which creates favourable conditions for karst development. The burial depth of the cave section exceeds 140 m, and the surface terrain is steep. These factors made it difficult to accurately identify hidden folds and structural planes during the exploration phase. At the same time, determining the exact location, shape, size, and scale of individual karst caves by the existing investigation methods is also difficult because of the concealment, complexity, and high heterogeneity of karst development.

#### Precipitation replenishment and groundwater impact

The engineering area is located on the Northern Guizhou Plateau and is part of the Daluo Mountains. The tunnel crosses a watershed within a horizontal circulation area with a large catchment area. The groundwater in the area is large and active. In the early surveying stage, the effect of seasonal rainfall on the groundwater recharge was not fully considered, which led to a misjudgment of the groundwater activities. Strengthening the backfill and tunnel structure is necessary, considering the erosion effect of groundwater on the tunnel structure during the rainy season.

The complex regional structure, precipitation replenishment and groundwater impact are the potential reasons contributing to the occurrence of large semi-filled karst cave tunnels in this case. It is advisable to monitor these three aspects for similar large karst tunnels.

## Full-life design of tunnel

### Crossing scheme in construction stage

The top of the cave intrudes into the boundary of the tunnel. Considering that roof-cutting construction is prone to collapse, the roof must be supported first. Based on our geological exploration results, the cave filling is poor. The vertical drop is large and there is a steep slope at the bottom, so the foundation of the supporting wall must be strengthened. When backfilling, the original karst river channel should be fully preserved to ensure the flow of groundwater.

The detailed construction steps are as follows.

**Figure 12:**
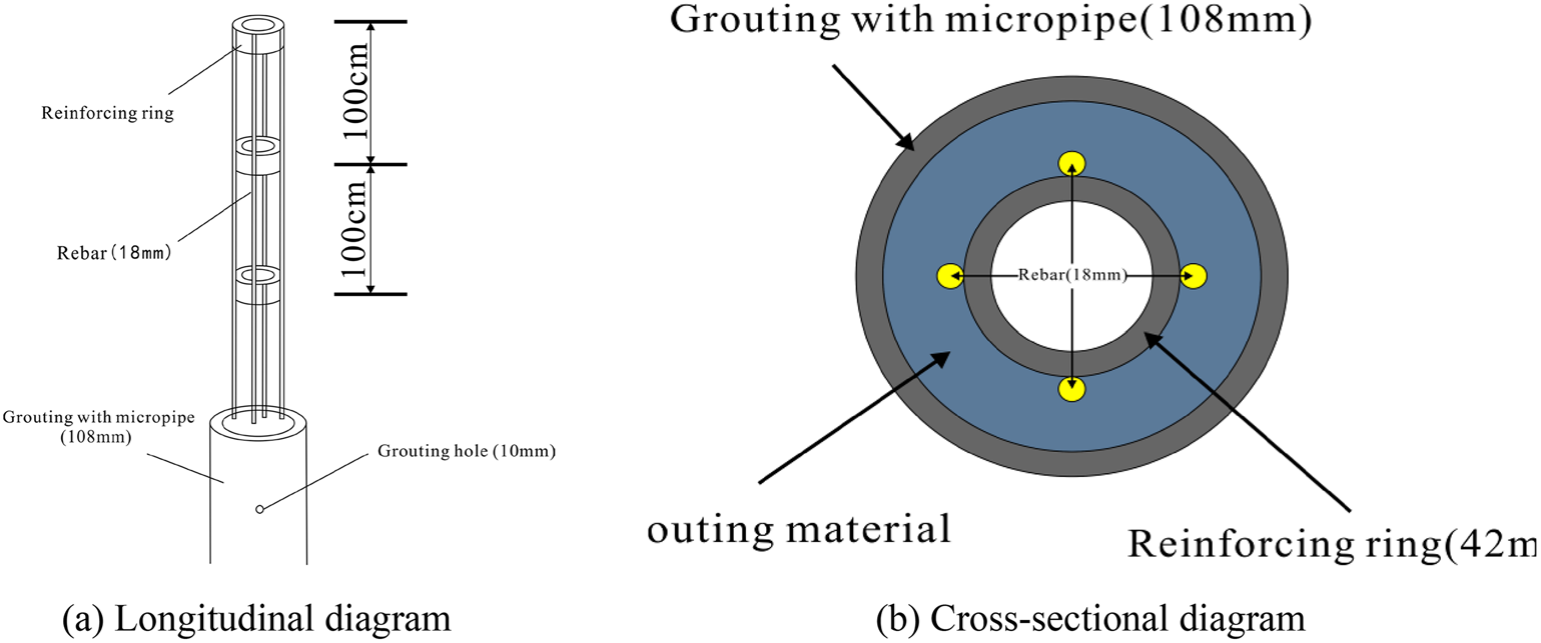
Steel pipe pile.

1. Backfilling of the karst cave: The bottom of the cave is filled with large-size block stones to form blind ditches. Groundwater can flow through these blind ditches to reduce the impact of tunnel excavation on the surrounding area. Based on the bottom block stone, the karst cave is filled to the bottom of the tunnel’s upper step with a layer of backfill soil.
2. Cast a supporting wall foundation: Φ108 steel pipe piles are used for the foundation. The steel pipe piles are embedded into stable bedrock for at least 1 m to ensure the stability of the supporting wall foundation. A reinforcement cage is set inside the pipe and grouted to reinforce the surrounding loose backfill soil and soft surrounding rocks, as shown in Figs. 12 and 13. The reinforcement in the steel pipe pile should be exposed for 0.3 m to connect with the ground beam above the foundation. The ground beam is arranged longitudinally along the line and serves as an expanded foundation for the supporting wall. It plays the role of connecting the supporting wall and the steel pipe pile foundation.

**Figure 13:**
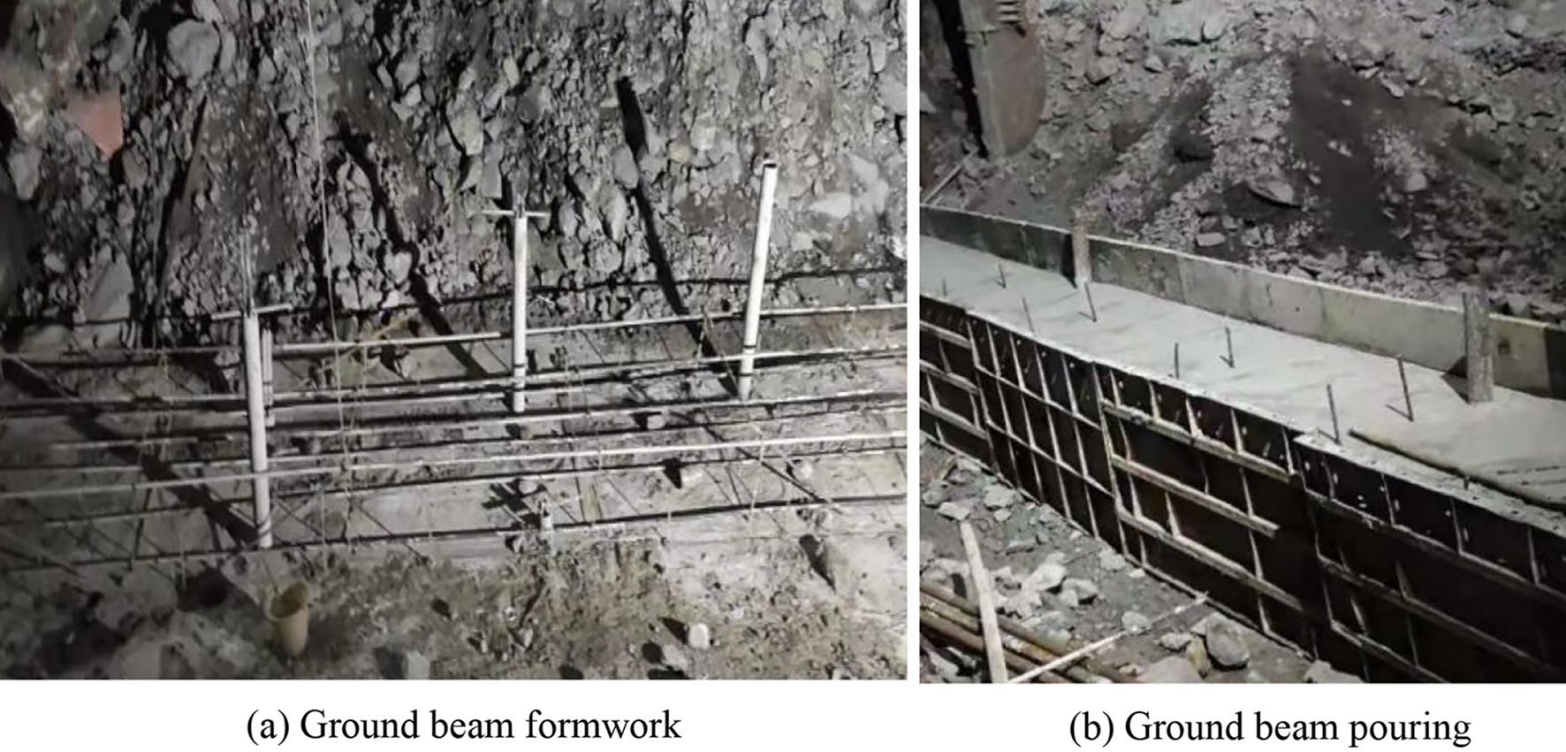
Ground beam.
3. Construct a supporting wall: The supporting wall has a section height of approximately 3–7 m and a width of 1 m. Settlement joints are established at intervals of 5 m along the longitudinal direction. Cushion blocks are employed at the top of the supporting wall to ensure proper contact with the cave roof. Figure 14 shows the supporting wall.

**Figure 14:**
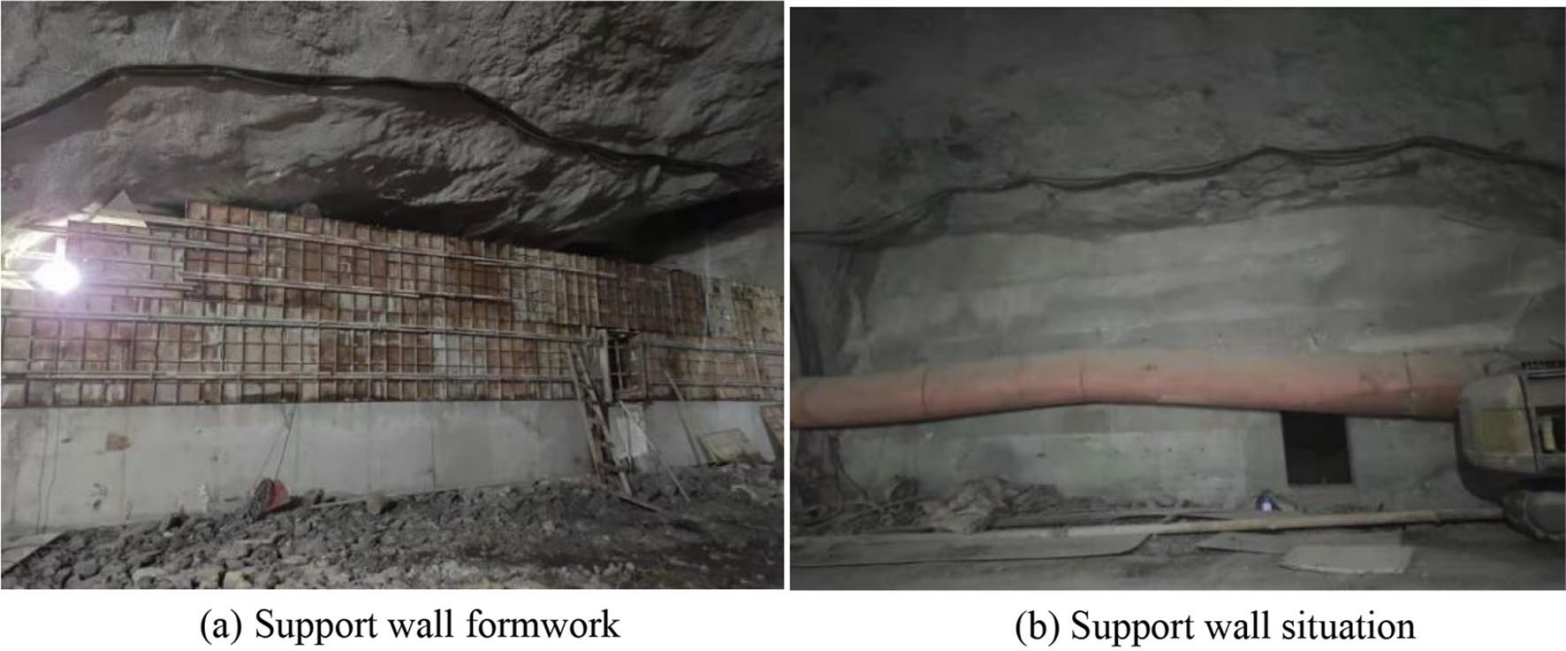
Supporting wall.
4. Drainage structure: The drainage structure is comprised of collecting wells and concrete drainage pipes. After the tunnel water is gathered from the left collection well, it is directed into the existing karst water channel from left to right through the drainage pipe beneath the tunnel. Figure 15 shows the drainage structure.

**Figure 15:**
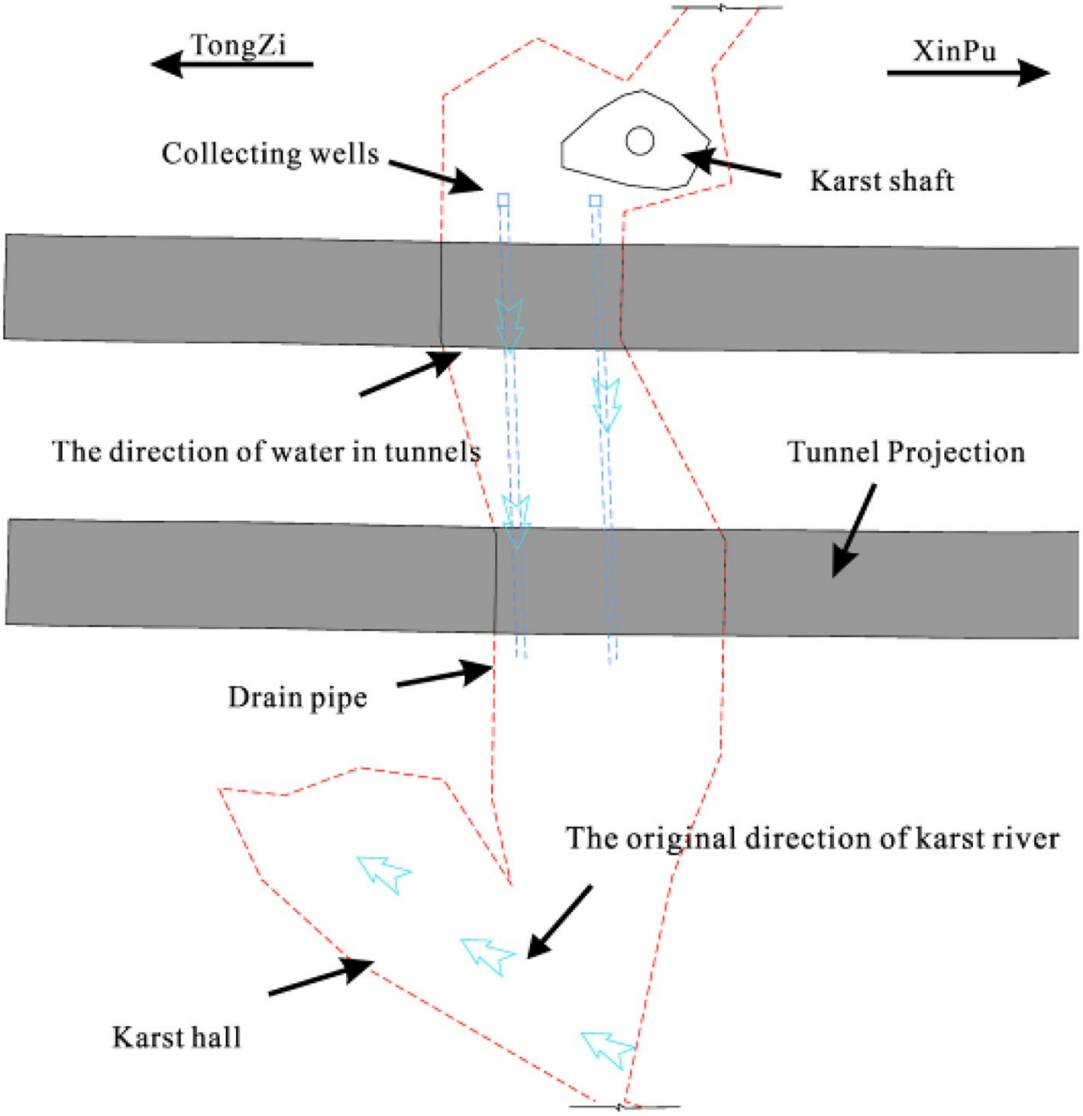
Drainage structure.

### Drainage and anti-floating in operation stage

#### Anti-floating reinforcement of ‘plate–pile–bedrock’

**Figure 16:**
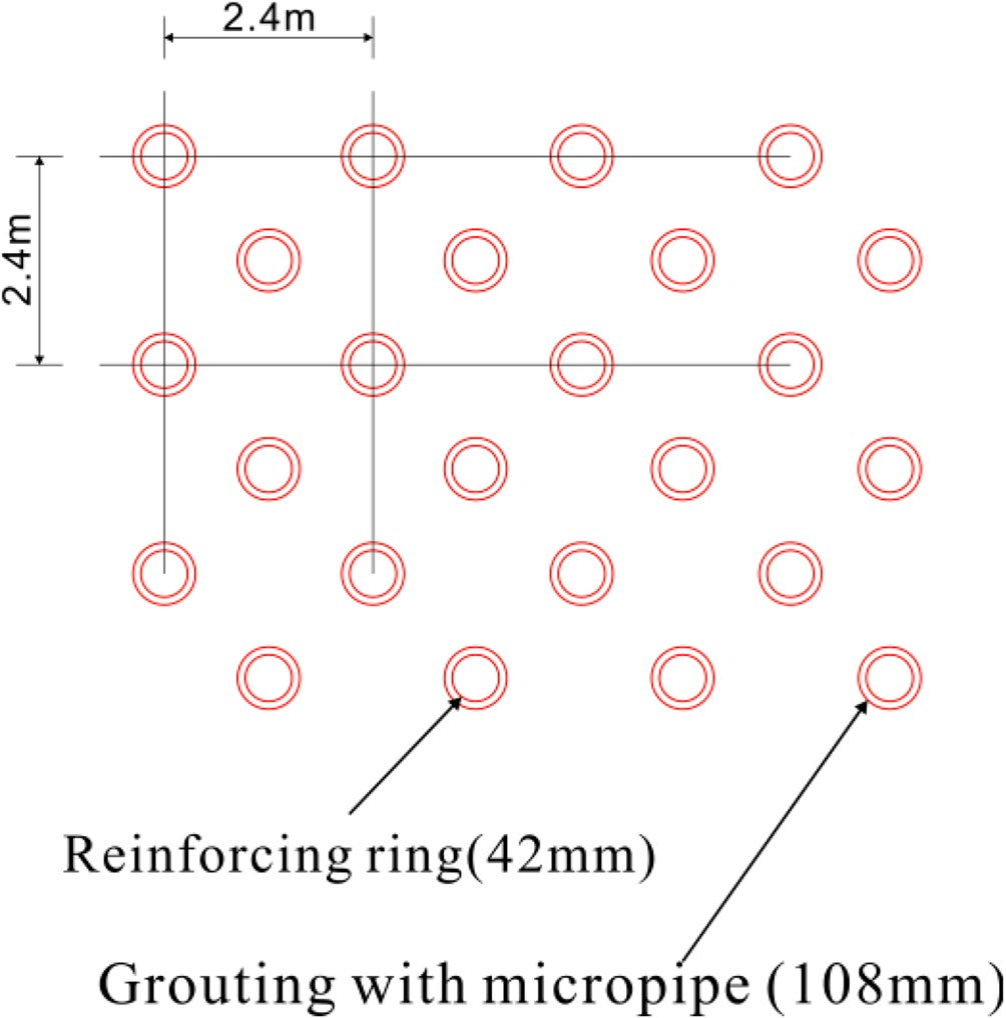
Plan of steel pipe pile.

**Figure 17:**
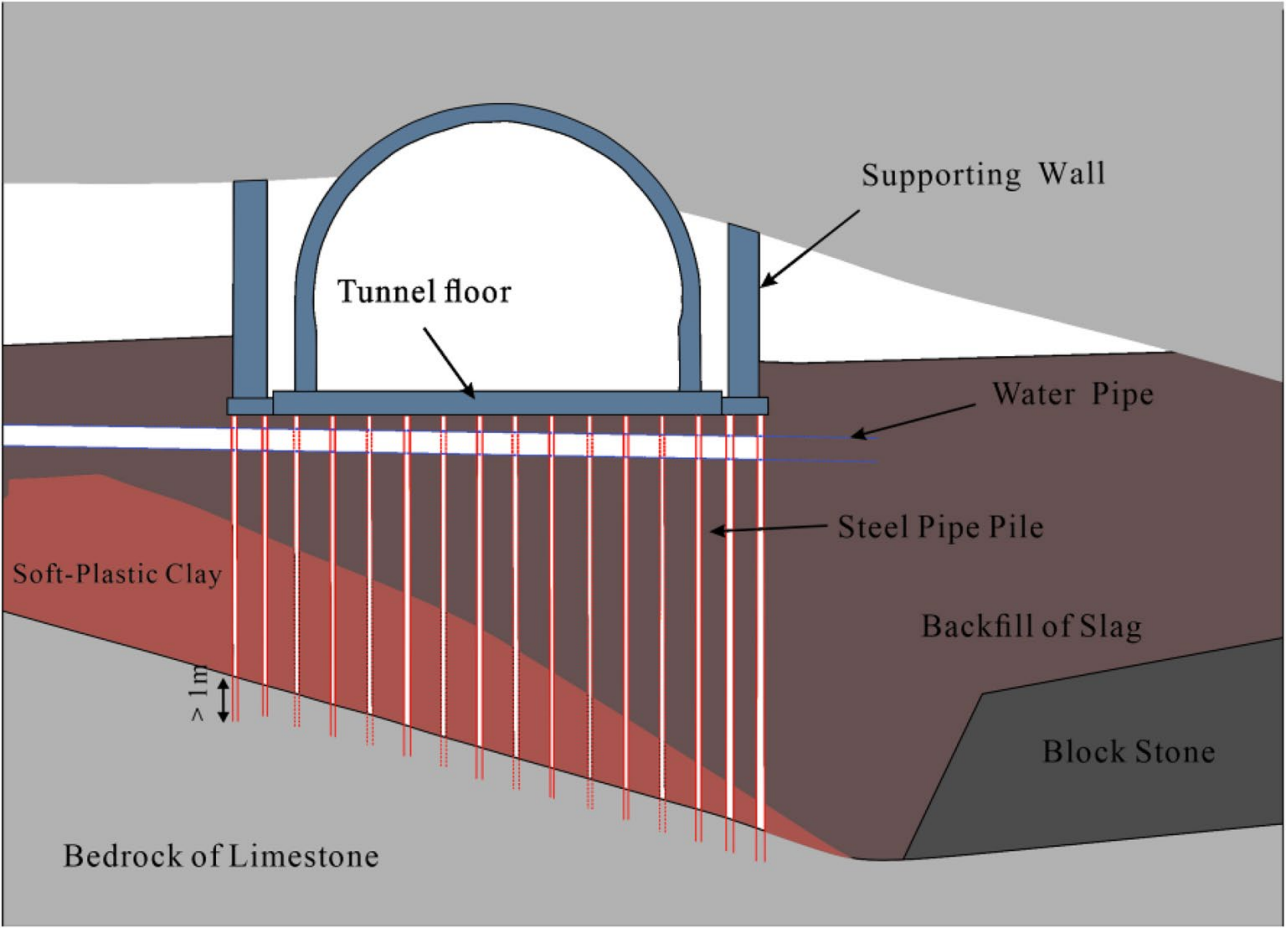
Diagram of 'plate–pile–bedrock' structure.

During tunnel construction, there is less underground river flow; nonetheless, the groundwater is greatly affected by seasonal precipitation. In future rainy periods, underground river flows may exceed design expectations. A large amount of groundwater can cause the collapse of backfill materials, leading to hazards such as tunnel floating and lining cracking. Therefore, reinforcing the backfill layer at the bottom of the tunnel with steel pipe piles is necessary. The steel pipe piles are arranged in the shape of 1.2 × 1.2 m plum blossoms, as shown in Fig. 16. The steel pipe pile embedded in the bedrock forms a ‘plate–pile–bedrock’ frame stress system with the tunnel floor. This system can realise the uniform transmission of the upper load and ensure the stability of the tunnel while reinforcing the filling body. The structural diagram of ‘plate–pile–bedrock’ is shown in Fig. 17.

#### A reverse drainage structure

There is seasonal water in the cave, which has a certain scouring effect on the tunnel lining and filling materials. During servicing periods, the filling material may flow with the groundwater, causing a blockage of the original karst pipeline. The blockage of the original drainage pipeline leads to an increase in water pressure after the lining, resulting in durability problems such as the collapse of the filling layer and cracking of the lining. To resolve the problem of the underground river not being discharged in time due to seasonal increases in water volume, we propose a new type of ‘bottom to top’ central ditch drainage structure. Two drainpipes with 180° elbows were buried in the left and right central ditches, respectively. Under normal conditions, the drainpipe would be set together with the inspection well. The so-called ‘bottom to top’ refers to the process of exploiting the high pressure accumulated at the bottom of the tunnel to pump out water automatically.

**Figure 18:**
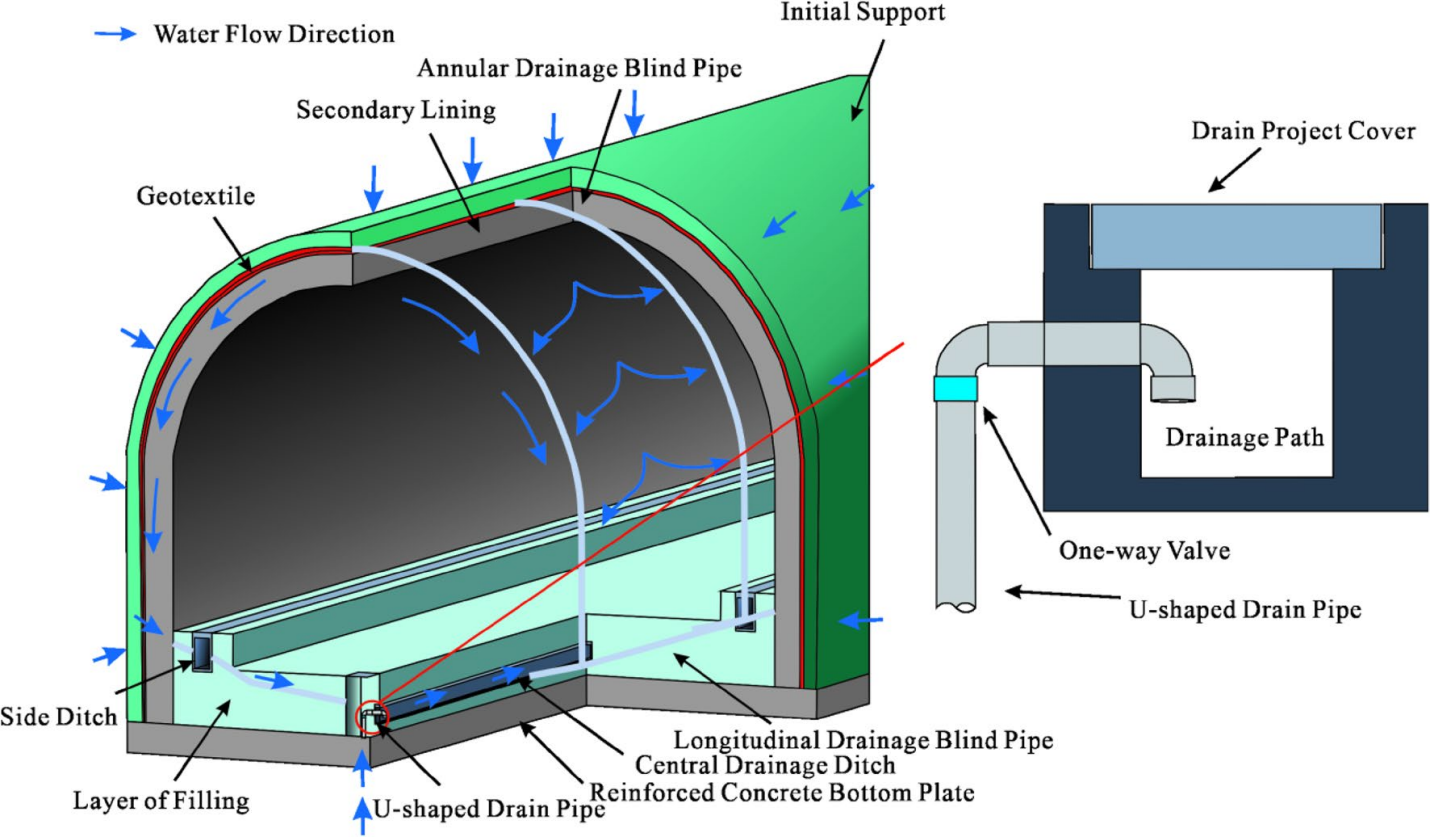
Working mechanism of drainage system.

Based on the original drainage structure of the tunnel, a U-shaped drainage pipe with a one-way valve was set at the central drainage ditch of the inspection well to form a ‘bottom to top’ reverse drainage structure. This structure can discharge unexpected groundwater in the tunnel from the central drainage ditch during the high-water period. The reverse drainage structure comprises a waterproof structure, a water diversion structure, and a drainage structure. The geotextile is arranged between the primary support and the secondary lining as a waterproof structure. While isolating part of the groundwater, the water that has invaded the secondary lining is discharged into the diversion system. It can filter particles in a portion of the water to ensure that the diversion structure is not blocked. The water diversion system comprises circumferential, longitudinal, and transverse drainage blind pipes. First, the water is collected into drainage ditches on both sides of the tunnel by circular drainage blind pipes. Then, the water in the side ditches is collected into the central drainage ditch through the longitudinal and transverse drainage blind pipes for drainage. During the dry season, the above structures meet the waterproofing and drainage requirements of the tunnel. Considering unexpected water scouring effects in the wet season, a U-shaped drainage pipe with a one-way valve was added to form a ‘bottom to top’ reverse drainage structure to meet the tunnel drainage and flotation resistance needs. Compared to traditional waterproofing and drainage structures, the ‘bottom to top’ reverse drainage structure automatically pumps water using the high pressure accumulated at the bottom of the tunnel. When excessive groundwater and water pressure accumulate at the tunnel bottom during the wet season, the water is discharged from bottom to top through the central drainage ditch and U-shaped drainage pipe. The working principle of the central drainage ditch with a U-shaped elbow is shown in Fig. 18.

## Effectiveness evaluation

**Figure 19:**
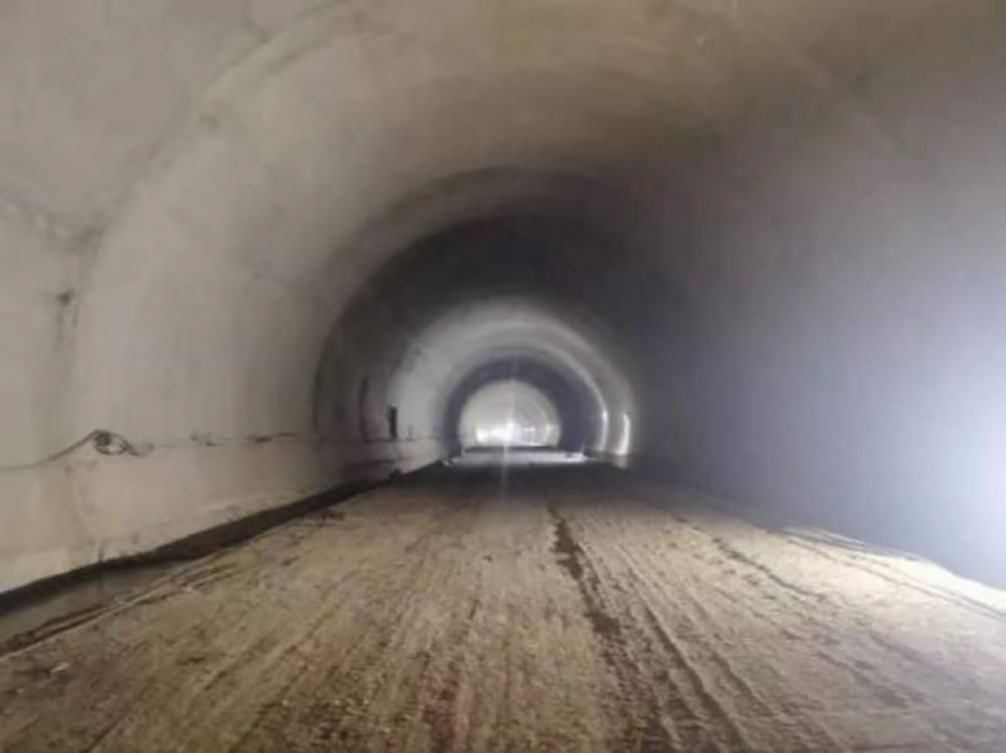
Lining situation.

Combined with the crossing and groundwater treatment methods in use at home and abroad, we proposed a full life cycle tunnel treatment method of ‘crossing in the dry season and an anti-floating drainage scheme in the wet season’. During construction, we adopted the top supporting scheme of ‘steel pipe pile–ground beam-supporting wall’, the filling layer reinforcement method of ‘plate–pile–bedrock’, and the flood discharge scheme of a central drainage ditch with U-shaped elbows. After the construction of the karst section was completed, the existing water was discharged automatically through the reserved channel and culvert pipe. Unexpected water that may occur during the use period can be discharged through the reserved central drainage ditch with U-shaped elbows. Besides, the tunnel lining has no leakage. Figure 19 shows the lining situation.

**Figure 20:**
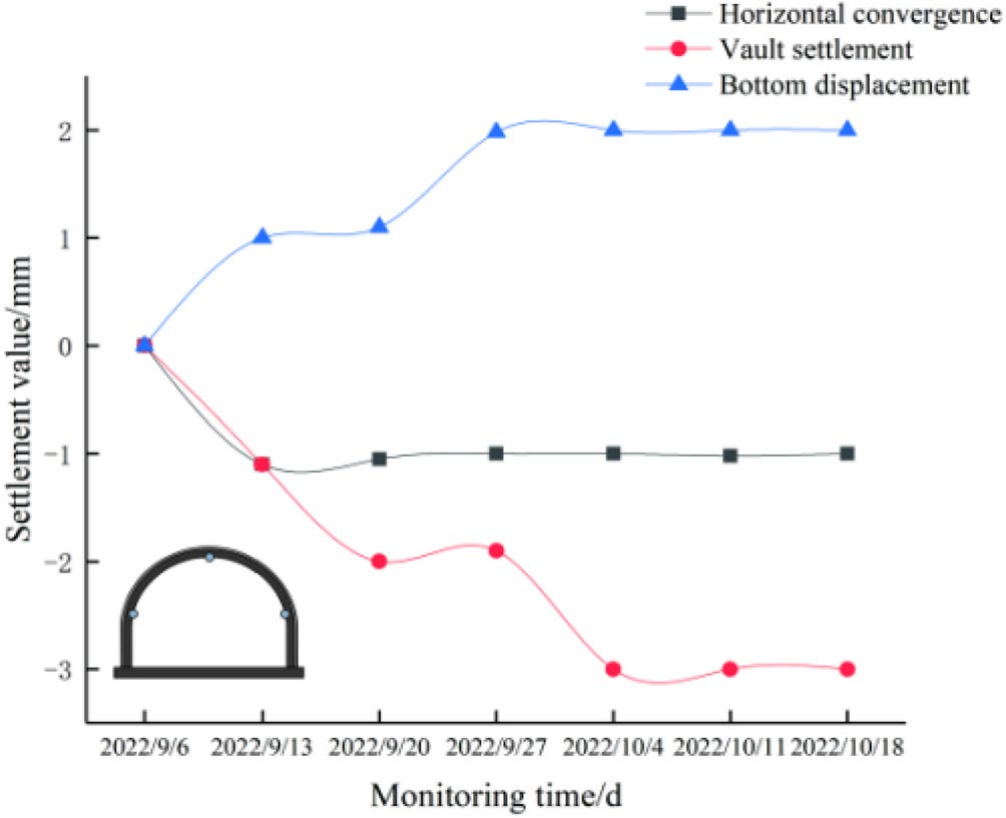
Time displacement curve of lining section.

After crossing the karst cave, we used the middle point stake number of the tunnel crossing the cave as the monitoring section. By monitoring the top settlement, bottom uplift, and horizontal convergence of the tunnel structure, we found that the top settlement of the tunnel was about 3 mm, the bottom uplift of the tunnel was about 2 mm, and the horizontal convergence of the side walls on both sides of the tunnel was about 1 mm, as shown in Fig. 20. Therefore, the displacement of the tunnel structure was within a fairly safe range. Considering any action of water beyond the expectation in the future flood season, pressure gauges and osmometers were set at the weak points of the lining to monitor the service performance of the structure.

## Summary and conclusions

Using the Huangchongyan Tunnel as an example, we propose a full life cycle disposal scheme for the safe passage of tunnels through karst caves. In this paper, we describe a construction scheme for crossing karst caves in the dry period and drainage and anti-floating in the wet period. We determined several conclusions and lessons.

1. Misjudging tunnels in large semi-filled karst caves is caused by several factors, including the complex regional structure, precipitation replenishment, and groundwater impact. For similar large karst tunnels, these three aspects should be monitored.
2. Compared to traditional treatment methods, crossing schemes for tunnels in large semi-filled karst caves with ‘steel pipe pile–ground beam-supporting walls’ have the advantages of preserving water pipelines, short construction periods, small investment, etc.
3. When the ‘plate–pile–bedrock’ stacking reinforcement method is adopted, the tunnel floor, steel pipe pile, and bedrock form a new frame force system. This system can effectively reduce the collapse problem caused by karst water erosion to the accumulation body. At the same time, the structure can help solve tunnel floating and lining cracking problems caused by confined water.
4. After crossing a large semi-filled karst cave using the ‘plate–pile–bedrock’ accumulation reinforcement method, the tunnel achieved good reinforcement effects. The displacement of the tunnel structure met safety requirements.
5. Considering unexpected water scouring effects in the wet season, a U-shaped drainage pipe with a one-way valve was added to form a ‘bottom to top’ reverse drainage structure to meet tunnel drainage and flotation resistance needs. Compared to traditional waterproofing and drainage structures, the ‘bottom to top’ reverse drainage structure automatically pumps water using the high pressure accumulated at the bottom of the tunnel.
6. The construction quality of the central drainage ditch with a U-shaped elbow is strictly required. Groundwater should be continuously pumped and drained to reduce the groundwater level during the construction of the drainage culvert, ground beams, tunnel floor, and pressure-resistant lining in the tunnel. Dry working conditions were provided for tunnel construction to ensure the quality of the concrete construction.

## Data Availability

All data generated or analysed during this study are included in this article.
